# Preoperative Neutrophil-to-Lymphocyte Ratio as a Predictor of Clinical Outcomes in Patients Undergoing Femoral Endarterectomy

**DOI:** 10.3390/jcm14010211

**Published:** 2025-01-02

**Authors:** Yohei Yamamoto, Ai Kazama, Toru Kikuchi, Toshifumi Kudo

**Affiliations:** Division of Vascular Surgery, Department of Cardiovascular Surgery, Institute of Science Tokyo, 1-5-45 Yushima, Bunkyo-ku, Tokyo 113-8519, Japan; kazama.srg2@tmd.ac.jp (A.K.); t.kikuchi.srg2@tmd.ac.jp (T.K.); toshikudo@gmail.com (T.K.)

**Keywords:** neutrophil-to-lymphocyte ratio, femoral endarterectomy, peripheral arterial disease, major adverse limb events, prognosis

## Abstract

**Background/Objectives:** This study aimed to evaluate the prognostic value of preoperative neutrophil-to-lymphocyte ratio (NLR) in patients with peripheral arterial disease (PAD) undergoing femoral endarterectomy. **Methods:** We performed a retrospective analysis of our institutional data, evaluating consecutive patients with PAD who underwent femoral endarterectomy between January 2013 and March 2023. The main objective was to assess the prognostic value of preoperative NLR for 5-year mortality. Additionally, we examined its relationship with perioperative clinicopathological features and 5-year major adverse limb events (MALEs). **Results:** During the study period, 200 consecutive patients underwent femoral endarterectomy. Of these, 128 patients with available NLR values within 30 days prior to surgery were analyzed. According to the receiver operating characteristic curve, the cut-off value of NLR was 4.0. Eighty-seven patients (68.0%) were assigned to the low-NLR group, and 41 patients (32.0%) to the high-NLR group. The frequency of postoperative complications did not differ significantly between the two groups. Freedom from MALEs up to five years was significantly lower in the high-NLR group (66.0% vs. 46.5%, *p* = 0.006). The overall survival rates were significantly lower in the high-NLR group (*p* < 0.001). At 1, 3, and 5 years, the survival rates in the low-NLR group were 96.4%, 91.6%, and 84.5%, respectively, while those in the high-NLR group were 84.2%, 59.5%, and 42.5%. Univariate analysis showed that cerebrovascular disease, end-stage renal disease, Rutherford category ≥ 4, a low albumin concentration (<3.5 g/dL), and a high NLR were significantly associated with 5-year mortality. Multivariate analysis indicated that a high NLR was the only independent factor associated with 5-year mortality. **Conclusions:** Preoperative NLR > 4.0 was significantly associated with 5-year rates of MALE and mortality in patients with symptomatic CFA occlusive disease who underwent femoral endarterectomy.

## 1. Introduction

Stenosis of the common femoral artery (CFA) is frequently observed in patients with peripheral arterial disease (PAD) and can represent a key lesion when treating symptomatic patients with multilevel disease. Although endovascular treatment (EVT) has become the first-line treatment for iliac and femoropopliteal lesions, surgical endarterectomy is still considered the gold standard for the treatment of CFA occlusive disease because of its established long-term outcomes [1]. Recently, several studies have suggested that advances in endovascular technology have made EVT a viable option for managing CFA lesions [2,3]. Therefore, the choice of surgical endarterectomy or EVT for CFA lesion is the upcoming debate. When determining management strategies, estimating a patient’s prognosis is crucial due to the significant variability in postoperative outcomes among patients undergoing lower-limb revascularization. Many laboratory markers have been investigated in PAD for diagnostic and prognostic purposes. However, there is no widely accepted method for predicting the prognosis of PAD patients, and most markers are not used in daily clinical practice [4].

The neutrophil-to-lymphocyte ratio (NLR) has been utilized as a novel prognostic indicator in various diseases. It is a simple biomarker for systemic inflammation that is convenient to apply in clinical practice [5]. Initially, the NLR was developed to evaluate sepsis and systemic infections. Subsequently, it has been shown to be useful in predicting prognosis and surgical outcomes in various cancers. However, its prognostic role in patients with PAD who are considered for lower-limb revascularization has not been well studied. In the present study, we evaluated the prognostic value of preoperative NLR in patients with PAD undergoing femoral endarterectomy.

## 2. Materials and Methods

This was a single-center, retrospective study. The clinical data of consecutive patients who underwent femoral endarterectomy at Tokyo Medical and Dental University Hospital (currently Institute of Science Tokyo Hospital) between January 2013 and March 2023 were collected.

During this period, 200 consecutive patients underwent femoral endarterectomy. Of these, 128 patients with available NLR values within 30 days prior to surgery were analyzed.

**Figure 1:**
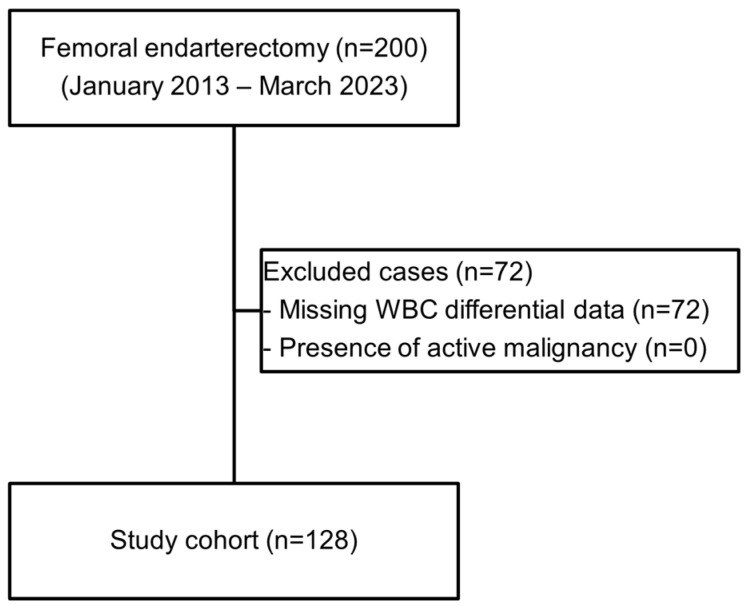
Flowchart of patient inclusion and exclusion criteria. WBC, white blood cell.

These included seven patients with a white blood cell count of over 10 × 10^9^/L. Patients with active malignancy were not included (Figure 1).

The NLR was calculated by dividing the absolute neutrophil count by the absolute lymphocyte count. Data on patient demographics, comorbidities, perioperative variables, and related clinical outcomes were extracted from electronic medical records. Coronary artery disease was defined as a history of angiographically documented coronary artery stenosis, percutaneous coronary intervention, or coronary artery bypass grafting. Routine coronary angiography was not performed in patients with PAD at our institution. Cerebrovascular disease was defined as a history of transient ischemic attack, stroke, or carotid revascularization. This study adhered to the ethical principles of the Declaration of Helsinki. The ethics committee of Institute of Science Tokyo Hospital approved this study (Reference number: M2023-307).

During the study period, femoral endarterectomy was performed on patients with PAD who were indicated for lower-limb revascularization and had >50% stenosis of the CFA. Adjunctive procedures for inflow and/or outflow lesions, such as concomitant EVT or bypass surgery, were performed based on the patient’s arterial anatomy and at the surgeon’s discretion.

In the absence of specific contraindications, an antiplatelet was prescribed and continued after surgery. Whether to add a second antithrombotic drug or a statin was determined based on a patient’s risk factors. The patients underwent clinical evaluation, including measurement of the ankle–brachial pressure index (ABI) at 3, 6, and 12 months, and a duplex ultrasound examination at 6 and 12 months. Follow-up was conducted every three to six months thereafter, with clinical assessment including ABI measurement or a duplex ultrasound examination. The main objective of this study was to assess the prognostic value of preoperative NLR for 5-year mortality. Additionally, we examined its relationship with perioperative clinicopathological features and 5-year major adverse limb events (MALEs). MALE was defined as ipsilateral major amputation or any ipsilateral reintervention.

### Statistical Analyses

A receiver operating characteristic (ROC) curve was performed to identify the optimal cut-off value of the NLR to predict mortality. Continuous variables were expressed as the mean ± standard deviation or median (interquartile range). The clinical characteristics of the two groups were compared using the χ^2^ test or Fisher’s exact test for categorical data and the Mann–Whitney U test for continuous data. The freedom from MALE and overall survival rates were analyzed using the Kaplan–Meier method. Cox proportional hazards regression analysis was used to identify the factors affecting mortality. Univariate screening with *p* < 0.20 was utilized, followed by multivariate analyses to identify independent factors for mortality. All statistical analyses were performed using BellCurve for Excel (Social Survey Research Information Co., Ltd., Tokyo, Japan).

## 3. Results

One hundred twenty-eight consecutive patients treated with femoral endarterectomy and who had available NLR values within 30 days prior to surgery were analyzed. Table 1 presents the patients’ demographic factors and preoperative comorbidities. Eighty-seven patients (68.0%) were men, and the mean age was 71.5 ± 8.25 years. The mean value of preoperative NLR was 4.13 ± 4.11. The most prevalent comorbidity was hypertension (85.2%), followed by dyslipidemia (73.4%). There were 35 patients (27.3%) with end-stage renal disease receiving chronic hemodialysis and 48 patients (37.5%) with chronic limb-threatening ischemia (CLTI), defined as Rutherford category ≥ 4. The distribution of disease severity among patients with CLTI, based on the Rutherford classification, was as follows: category 4 (*n* = 20), category 5 (*n* = 27), and category 6 (*n* = 1). There were five patients with CLTI who had foot infections, and all of them had a white blood cell count of over 10 × 10^9^/L.

**Figure 2:**
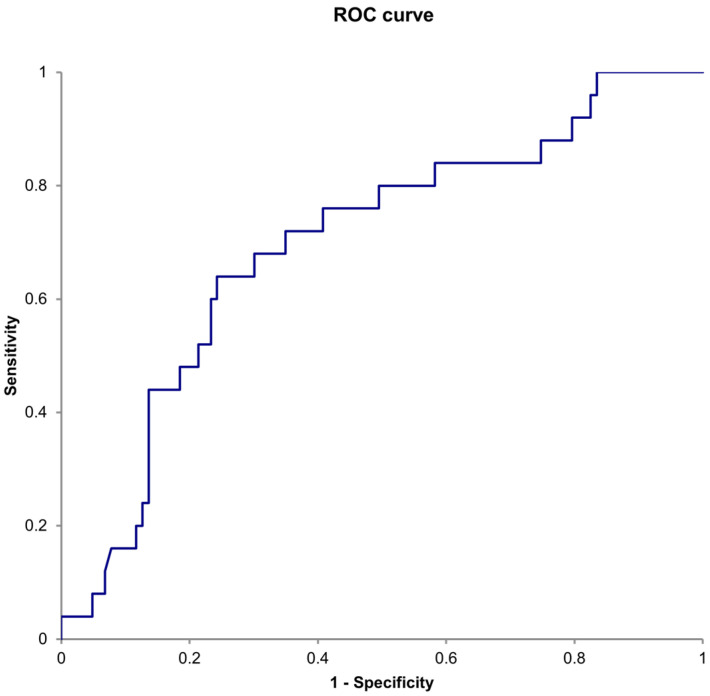
Receiver operating characteristic (ROC) curve showing preoperative neutrophil-to-lymphocyte ratio for predicting 5-year mortality.

The median follow-up period was 30.5 (13–67.25) months. Twenty patients (15.6%) developed postoperative complications within 30 days, and thirty patients (23.4%) died within five years after surgery. The ROC curve showed that the area under the curve (AUC) for the NLR in predicting 5-year mortality was 0.698 (95% CI, 0.582–0.813; *p* < 0.001), with a cut-off value of 4.0 (sensitivity, 64.0%; specificity, 75.7%) (Figure 2).

Based on the cut-off value of the NLR, 87 patients (68.0%) were assigned to the low-NLR group (≤4.0), and the remaining 41 patients (32.0%) were assigned to the high-NLR group (>4.0).

Comparisons between the low- and high-NLR groups are shown in Table 2. The proportion of patients with end-stage renal disease, non-ambulatory status, and CLTI was higher in the high-NLR group than in the low-NLR group.

The frequency of postoperative complications did not differ significantly between the two groups (Table 3).

**Figure 3:**
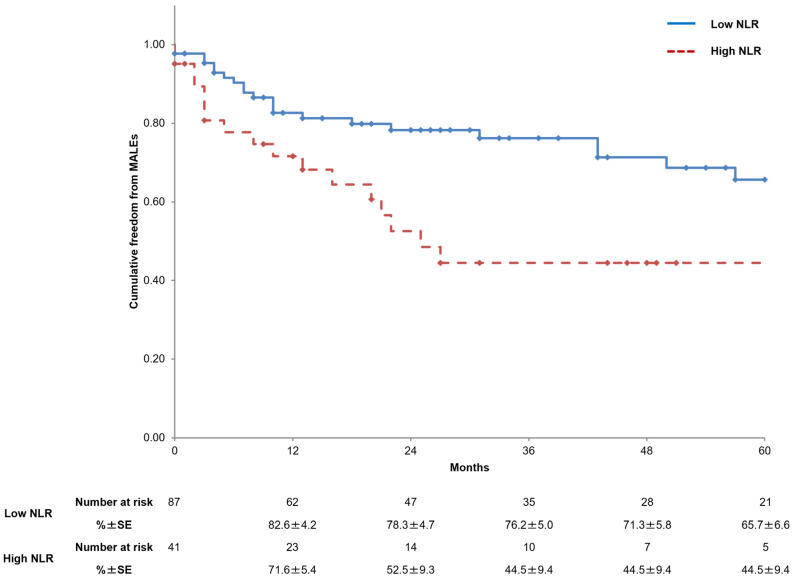
Kaplan–Meier curves showing freedom from major adverse limb events (MALEs) in the two study groups. NLR, neutrophil-to-lymphocyte ratio; SE, standard error.

Freedom from MALEs up to five years was significantly lower in the high-NLR group (66.0% vs. 46.5%, *p* = 0.006) (Figure 3).

The overall estimated survival rates for all subjects at 1, 3, and 5 years were 92.6%, 82.0%, and 71.3%, respectively. The causes of death were cardiovascular diseases (*n* = 15, 50.0%), malignancy (*n* = 4, 13.3%), infectious diseases (*n* = 4, 13.3%), interstitial pneumonia (*n* = 1, 3.3%), and unknown causes (*n* = 6, 20.0%). For all patients who died from malignancy, the disease was either diagnosed after surgery or represented a recurrence following clinical remission. Cardiovascular death was significantly more frequent in the high-NLR group (*n* = 9) compared to the low-NLR group (*n* = 6) (22.0% vs. 6.9%; *p* = 0.014).

**Figure 4:**
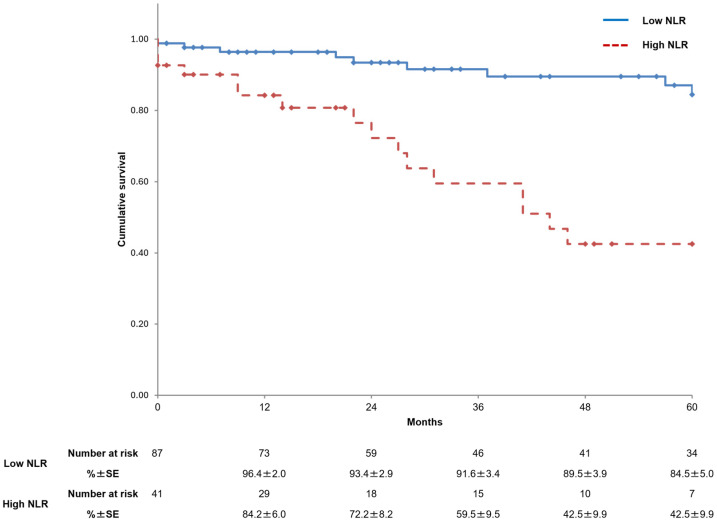
Kaplan–Meier curves showing survival in the two study groups. NLR, neutrophil-to-lymphocyte ratio; SE, standard error.

In the low-NLR group, the estimated survival rates at 1, 3, and 5 years were 96.4%, 91.6%, and 84.5%, respectively, while those for the same periods in the high-NLR group were 84.2%, 59.5%, and 42.5%, respectively (*p* < 0.001) (Figure 4).

Univariate analysis showed that a high NLR, a low albumin concentration (<3.5 g/dL), cerebrovascular disease, end-stage renal disease, and CLTI were significantly associated with 5-year mortality. Multivariate analysis indicated that a high NLR was the only independent factor associated with 5-year mortality (Table 4).

## 4. Discussion

The present study investigated the predictive factors for long-term outcomes in symptomatic PAD patients undergoing femoral endarterectomy, focusing on preoperative NLR. The results from our study indicated that a preoperative NLR > 4.0 was significantly associated with the incidence of MALEs and 5-year mortality following femoral endarterectomy, although an elevated white blood cell count alone was not a predictor of long-term outcomes in these patients. Kaplan–Meier survival analysis demonstrated that the difference in 5-year mortality between the low- and high-NLR groups was more pronounced than the difference in the incidence of MALEs. Potential mechanisms for the observed results include systemic inflammation and hypercoagulability, both of which are major risk factors for cardiovascular events. The present study also demonstrated that serum albumin levels were significantly lower in the high-NLR group. This finding is consistent with the role of serum albumin as a marker reflecting not only a patient’s nutritional status but also their inflammatory status [6].

Most studies on femoral endarterectomy have focused on technical aspects [7], postoperative complications [8], vessel patency, and comparisons of results with EVT [2]. Although the prognosis of patients with symptomatic CFA occlusive disease and its associated factors has not been well studied, it is well known that CLTI is a poor prognostic factor [9,10]. Notably, the analysis in the present study demonstrated that a high NLR had a stronger association with 5-year mortality than CLTI.

Currently, EVT is commonly used for iliac and superficial femoral artery lesions. However, it is still less favored for CFA lesions due to its reduced patency, the risk of occlusion of the profunda femoris artery, and the risk of stent fracture. The advantages of EVT are its low procedural invasiveness and lower rate of postoperative complications. In addition, a recent study demonstrated that EVT using stenting exhibits similar 2-year patency rates compared to endarterectomy [2]. Other recent studies suggest that EVT is suitable for a particular group of patients with symptomatic CFA occlusive disease, based on analyses of lesion complexity [3] and short-term results [11].

Thus, EVT can be the treatment of choice for selected patients with CFA occlusive disease, and the indications for treatment are likely to evolve.

In addition to lesion assessment and short-term results, a simple and reliable method for predicting the prognosis of patients with CFA occlusive disease could also contribute to determining whether to select endarterectomy or perform EVT.

The NLR was first proposed by Zahorec in 2001 as a parameter of systemic inflammation in critically ill patients [12]. Today, the NLR is widely used as an indicator of disease activity, severity, and prognosis in patients with infectious diseases [13], severe trauma [14], or cancers [15]. Additionally, the NLR has been studied as a clinically useful marker in cardiovascular diseases, particularly in coronary artery disease [16,17] and cerebrovascular disease [18,19].

Although the utility of the NLR in patients with PAD has not been established, the number of studies focusing on this topic has been increasing in recent years [20].

The NLR has been reported to be associated with disease severity and prognosis in patients with PAD [21,22]. Several previous studies have demonstrated the predictive power of the NLR for postoperative outcomes following lower-limb revascularization.

Bath et al. reported that a high preoperative NLR was associated not only with disease severity but also with postoperative complications in patients with PAD undergoing revascularization [23]. In 2018, Pourafkari et al. conducted a large retrospective cohort study and reported that the NLR was an independent predictor of MALEs and 10-year mortality in patients with PAD undergoing revascularization [24].

González-Fajardo et al. investigated patients with CLTI and demonstrated that an NLR > 5 was independently associated with poor 5-year amputation-free survival after infrainguinal revascularization [25]. Similarly, Chan et al. focused exclusively on patients with CLTI undergoing infrapopliteal EVT and found that an NLR ≥ 5.25 was associated with nearly double the risk of 12-month mortality [26].

In the present study, the incidence of early postoperative complications was not statistically different between the low- and high-NLR groups. This suggests that femoral endarterectomy should not be avoided in patients with a high NLR due to concerns about postoperative complications. However, our results show that patients with a high NLR had significantly poorer long-term outcomes, suggesting that in these patients, minimally invasive approaches and short-term outcomes may be prioritized over the long-term durability of treatment effects. In other words, patients with a high NLR may be better candidates for EVT, whereas patients with a low NLR are more likely to benefit from the long-term durability of endarterectomy.

The preoperative estimation of prognosis in patients with PAD is important not only for determining the revascularization strategy but also for planning the postoperative follow-up strategy. The main causes of death in patients with PAD are cardiovascular diseases [27].

In the present study, half of the deaths were due to cardiovascular causes. This result is consistent with a large previous study that focused on the causes of death among patients with symptomatic PAD [28]. Moreover, the incidence of cardiovascular deaths was significantly higher in the high-NLR group, and the proportion of cardiovascular deaths among all deaths in the high-NLR group was 56.3% (9/16).

These findings suggest another important factor to consider for improving the therapeutic outcomes of patients with symptomatic CFA occlusive disease.

To maximize the long-term durability of surgical endarterectomy, meticulous follow-up—including strict surveillance and optimal medical therapy to reduce cardiovascular risk—should be implemented, particularly for patients with a high NLR.

This study had certain limitations, such as its retrospective nature and a relatively small sample size. Participants in this study were limited to individuals with symptomatic CFA occlusive disease, and the cut-off value for the NLR used in this study was not consistent with that in other relevant studies. The lack of a standardized NLR cut-off across studies limits the generalizability of our findings to broader patient populations. Thus, further studies are warranted to standardize the utility of the NLR in patients with PAD.

## 5. Conclusions

Preoperative NLR > 4.0 was significantly associated with 5-year rates of MALE and mortality in patients with symptomatic CFA occlusive disease who underwent femoral endarterectomy. The NLR could serve as a helpful prognostic marker to guide clinical decision-making in patients with PAD.

## Figures and Tables

**Table 1:** Demographics and preoperative comorbidities of the study population.

	All Patients (*n* = 128)
Age, years	71.5 ± 8.25
Men	87 (68.0)
Neutrophil-to-lymphocyte ratio	4.13 ± 4.11
Serum albumin (g/dL)	3.81 ± 0.59
Statin use	80 (62.5)
Hypertension	109 (85.2)
Dyslipidemia	94 (73.4)
Diabetes mellitus	82 (64.1)
Coronary artery disease	54 (42.2)
Cerebrovascular disease	26 (20.3)
COPD	20 (15.6)
End-stage renal disease	35 (27.3)
Ever smoked	100 (78.1)
Non-ambulatory status	8 (6.3)
Rutherford classification	
3	80 (62.5)
4	20 (15.6)
5	27 (21.1)
6	1 (0.8)

Data are expressed as mean ± standard deviation or number (percentage). COPD, chronic obstructive pulmonary disease.

**Table 2:** Comparison between the low and high neutrophil-to-lymphocyte ratio (NLR) groups.

	Low NLR (*n* = 87)	High NLR (*n* = 41)	*p*-Value
Age, years	71.8 ± 8.29	71.4 ± 8.22	0.784
Men	54 (62.1)	33 (80.5)	0.037
NLR	2.46 ± 0.76	7.65 ± 5.76	<0.001
Serum albumin (g/dL)	3.98 ± 0.50	3.46 ± 0.63	<0.001
Statin use	56 (64.4)	24 (58.5)	0.525
Hypertension	72 (82.8)	37 (90.2)	0.302
Dyslipidemia	65 (74.7)	29 (70.7)	0.634
Diabetes mellitus	55 (63.2)	27 (65.9)	0.772
Coronary artery disease	35 (40.2)	19 (46.3)	0.514
Cerebrovascular disease	15 (17.2)	11 (26.8)	0.208
COPD	13 (14.9)	7 (17.1)	0.757
End-stage renal disease	13 (14.9)	22 (53.7)	<0.001
Ever smoked	69 (79.3)	31 (75.6)	0.637
Non-ambulatory status	2 (2.3)	6 (14.6)	0.013
CLTI	23 (26.4)	25 (61.0)	<0.001

Data are expressed as mean ± standard deviation or number (percentage). NLR, neutrophil-to-lymphocyte ratio; COPD, chronic obstructive pulmonary disease; CLTI, chronic limb-threatening ischemia.

**Table 3:** Comparison of the intraoperative and early postoperative outcomes between the low and high neutrophil-to-lymphocyte ratio (NLR) groups.

	Low NLR (*n* = 87)	High NLR (*n* = 41)	*p* Value
Simple endarterectomy	20 (23.0)	12 (29.3)	0.444
Endarterectomy + inflow procedure	45 (51.7)	16 (39.0)	0.180
Endarterectomy + outflow procedure	34 (39.1)	22 (53.7)	0.121
Complications within 30 days	12 (13.8)	8 (19.5)	0.406
Cardiac failure	0 (0.0)	1 (2.4)	
Cerebrovascular complications	1 (1.1)	3 (7.3)	
Wound complications	4 (4.6)	0 (0.0)	
Bleeding	5 (5.7)	3 (7.3)	
Major amputation	1 (1.1)	1 (2.4)	
Ischemic colitis	1 (1.1)	0 (0.0)	
Death within 30 days	1 (1.1)	2 (4.9)	0.240
Postoperative hospital stay (days)	9 (8–13.5)	12 (7–30.25)	0.034

Data are expressed as number (percentage) or median (interquartile range). NLR, neutrophil-to-lymphocyte ratio.

**Table 4:** Univariate and multivariable analyses of predictors of 5-year overall mortality.

	Univariate Analysis		Multivariate Analysis	
Risk Factor	HR (95% CI)	*p*-Value	HR (95% CI)	*p*-Value
Age	1.01 (0.96–1.06)	0.715		
WBC count > 10 × 10^9^/L	2.02 (0.47–8.58)	0.343		
NLR > 4.0	5.24 (2.31–11.93)	<0.001	2.60 (1.05–6.45)	0.039
Albumin < 3.5 g/dL	5.17 (2.33–11.43)	<0.001	2.08 (0.83–5.20)	0.119
Hypertension	5.30 (0.72–39.17)	0.103	4.38 (0.53–35.98)	0.169
Dyslipidemia	0.79 (0.34–1.84)	0.592		
Diabetes mellitus	1.68 (0.67–4.22)	0.266		
Coronary artery disease	1.14 (0.52–2.51)	0.746		
Cerebrovascular disease	2.77 (1.26–6.10)	0.011	1.11 (0.45–2.74)	0.813
COPD	1.22 (0.48–3.06)	0.676		
End-stage renal disease	5.34 (2.36–12.11)	<0.001	1.56 (0.57–4.28)	0.385
Ever smoked	1.36 (0.47–3.97)	0.572		
Non-ambulatory status	1.70 (0.40–7.24)	0.472		
CLTI	4.52 (1.95–10.51)	<0.001	2.41 (0.87–6.65)	0.091
Statin use	0.62 (0.28–1.37)	0.238		

HR, hazard ratio; CI, confidential interval; WBC, white blood cell; NLR, neutrophil-to-lymphocyte ratio; COPD, chronic obstructive pulmonary disease; CLTI, chronic limb-threatening ischemia.

## Data Availability

The data that support the findings of this study are available from the corresponding author.
